# Mitral valvuloplasty with left atrial appendage closure and pacemaker implantation in a dog with severe myxomatous mitral valve degeneration: a case report

**DOI:** 10.1186/s12917-022-03284-7

**Published:** 2022-05-16

**Authors:** Tomohiko Yoshida, Ryosuke Namiki, Katsuhiro Matsuura

**Affiliations:** 1 VCA Japan Shiraishi Animal Hospital, Saitama, 350-1304 Japan; 2 Department of Veterinary Surgery, Faculty of Veterinary Medicine, Tokyo University of Agriculture and Technology, 183-8509 Tokyo, Japan

**Keywords:** Mitral valve repair, Sick sinus syndrome, Epicardial left atrial appendage closure device

## Abstract

**Background:**

Mitral valvuloplasty (MVP) has been widely recognized as a treatment option for myxomatous mitral valve disease (MMVD). However, postoperative complications such as thromboembolism, arrhythmia, and pancreatitis in some cases have resulted in death. We treated a dog with severe MMVD complicated by impaired sinus function with MVP and pacemaker implantation. Also, due to an intrinsic procoagulant state and severe arrhythmia after the MVP, left atrial appendage (LAA) closure was performed to reduce the postoperative risk of thrombosis.

**Case presentation:**

An 11-year-old castrated 7.5-kg male Miniature Schnauzer with a history of congestive heart failure was brought to Shiraishi Animal Hospital for MMVD surgical treatment. Echocardiography revealed an enlarged left atrium and ventricle secondary to MMVD. Sinus arrest with 2 to 3-second periods of asystole was identified by electrocardiogram. Mitral valvuloplasty was performed with cardiopulmonary bypass to treat the MMVD. After coronary reperfusion, there was no spontaneous electrical activity. Cardiac arrest continued. Based on this surgical outcome, a permanent pacemaker was implanted. In addition, LAA closure with an AtriClip was performed to prevent intra-atrial thrombus formation. Cardiac remodeling and congestion were ameliorated after surgery. Sinus rhythm was restored 5 days postoperatively; however, the patient continued pacemaker dependent. All cardiac drugs were discontinued 3 months after surgery. The owner reported no postoperative complications (i.e., thrombosis), and the patient was brought for a check-up 4 months after the operation in good health.

**Conclusions:**

For surgical MMVD cases complicated with impaired sinus function, the chances of spontaneous sinus rhythm are low, requiring pacemaker implantation. LAA closure may be considered to protect against decreased atrial function after mitral valvuloplasty and prevent intra-atrial thrombus formation.

## Background

Myxomatous mitral valve disease (MMVD) is the most common acquired heart disease in dogs [1, 2]. Medical treatment can improve a dog’s clinical condition and prolong its lifespan with congestive heart failure, as shown in several recent clinical trials and studies [3, 4]. However, in MMVD cases with congestive heart failure prognosis may be poor [2]. For humans with congestive heart failure, medical care includes surgical mitral valvuloplasty and valve replacement to treat mitral regurgitation [5]. On the other hand, in small animal medicine, mitral valvuloplasty is often superior to valve replacement when considering the need for permanent antithrombotic therapy and the issues of biocompatibility of the latter [6]. Mitral valvuloplasty outcomes have improved in recent years, but postoperative complications such as thromboembolism, arrhythmia, and renal failure, in some cases, have resulted in death [7, 8]. In particular, the incidence of postoperative thrombosis and arrhythmia is very high and can be fatal [7–9]. A pacemaker is occasionally required for severe arrhythmia during surgery for valvular disease [10–12]. Since severe arrhythmia promotes thrombi, LAA closure is frequently needed to prevent thrombus formation in the left atrium [13, 14].

We performed mitral valvuloplasty for a case of severe MMVD accompanied by bradyarrhythmia. After the intracardiac procedure, a pacemaker was implanted to treat an emergency cardiac arrest. LAA closure with an AtriClip was also performed to reduce the postoperative risk of thrombosis.

## Case presentation

**Fig. 1:**
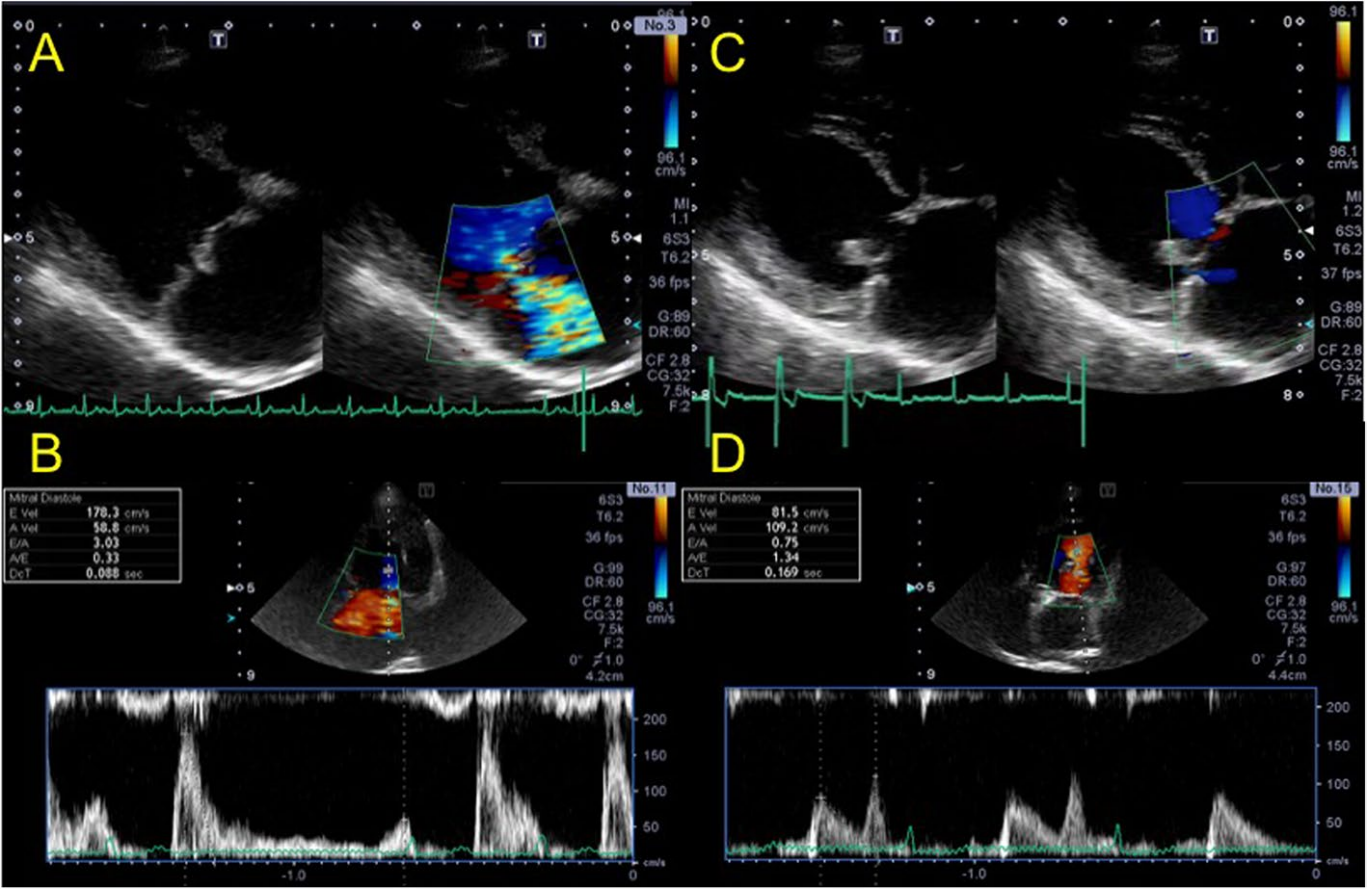
Transthoracic echocardiographic imaging before and after mitral valvuloplasty. **a** Right parasternal long-axis LV inflow view imaging before mitral valvuloplasty. Severe mitral regurgitation was observed. **b** E wave velocity gained from left parasternal apical four chamber view before mitral valvuloplasty. E wave increased (178 cm/sec) (**c**) Right parasternal long-axis LV inflow view imaging after mitral valvuloplasty. Mitral regurgitation mostly disappeared (**d**) E wave velocity gained from left parasternal apical four chamber view after mitral valvuloplasty. E wave decreased significantly compared with before (81.5 cm/sec)

An 11-year-old castrated male 7.5-kg Miniature Schnauzer was brought to Shiraishi Animal Hospital, Saitama, Japan, for surgical treatment of MMVD ACVIM stage C. Pimobendan (Vetmedin; Boehringer Ingelheim, Ingelheim, Germany, 0.5 mg/kg, BID), torasemide (Luprac; Mitsubishi Tanabe Pharma Corporation, Osaka, Japan, 0.25 mg/kg, BID), enalapril (Enacard; Boehringer Ingelheim, Ingelheim, Germany, 0.5 mg/kg, BID), and cilostazol (Cilostazol; Sawai Pharmaceutical Co., Ltd. Osaka, Japan,10 mg/kg, BID) due to history of pulmonary edema and syncope had been prescribed. The patient was in good health except for mild polydipsia. A grade IV/VI systolic murmur over the left cardiac apex was noted on presentation. Echocardiography revealed MMVD with prolapse of the anterior and posterior leaflets, severe left atrial enlargement (LA/Ao 2.84), a normalized left ventricular internal dimension in diastole (LVIDDN) of 2.37, and an elevated E velocity (178 cm/s) (Fig. 1). An electrocardiogram showed sinus arrest with a 2 to 3-second period of asystole. Biochemical parameters revealed increased blood urea nitrogen (38.2 mg/dl, reference range: 9.2–29.9 mg/dl) and decreased Na (132.4 mmol/L, reference range: 142–152 mmol/L), and Cl (103.9 mmol/L, reference range: 105–117 mmol/L). The patient was considered at high risk for pulmonary edema and progressive left-sided heart failure due to MMVD. A mitral valvuloplasty procedure and its associated risks were discussed with the owner. A pacemaker was prepared based on the patient’s clinical history.

**Fig. 2:**
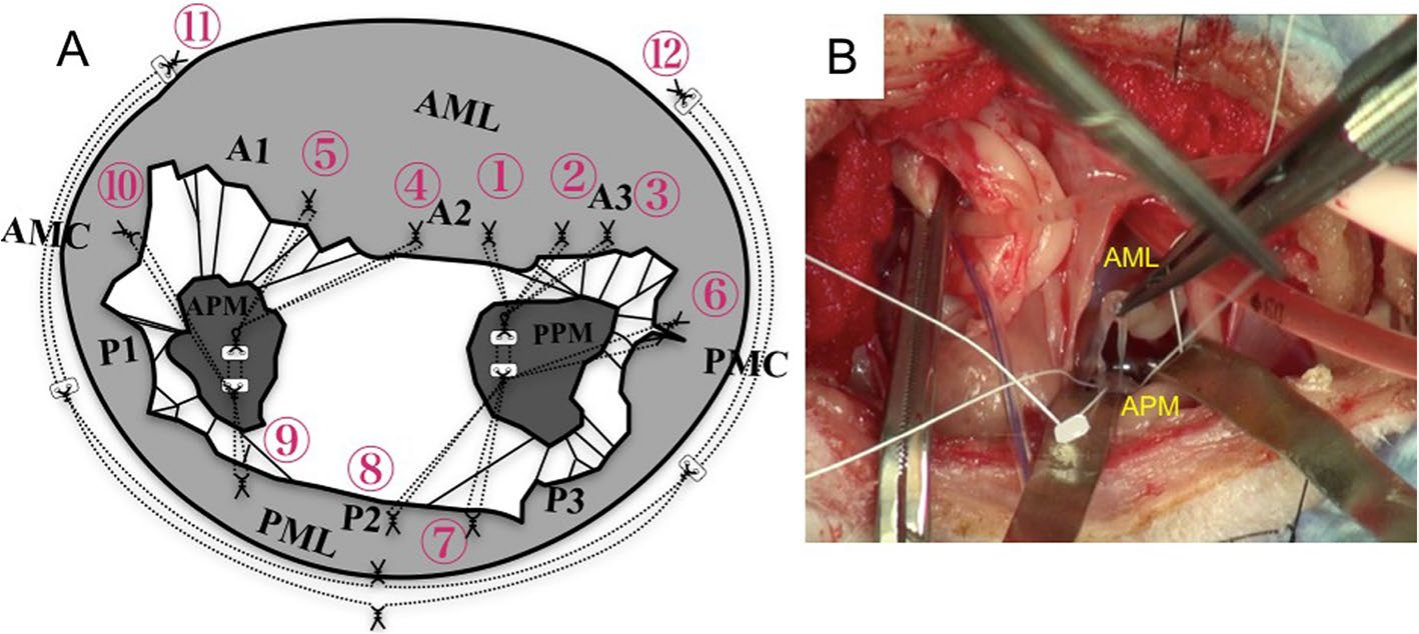
Illustration of surgical technique and intraoperative photograph. **a** The order of artificial chordae tendineae reconstruction and mitral annuloplasty. AML and PML were anchored by ePTFE suture of ①–⑩ through the APM and PPM. Suture annuloplasty was performed using ePTFE suture of ⑪–⑫. **b** This photo shows the artificial chordae tendineae reconstruction. AMC: anterior mitral commissure; AML: anterior mitral leaflet; APM: anterior papillary muscle; ePTFE: expanded polytetrafluoroethylene; PMC: posterior mitral commissure; PML: posterior mitral leaflet; PPM: posterior papillary muscle

**Fig. 3:**
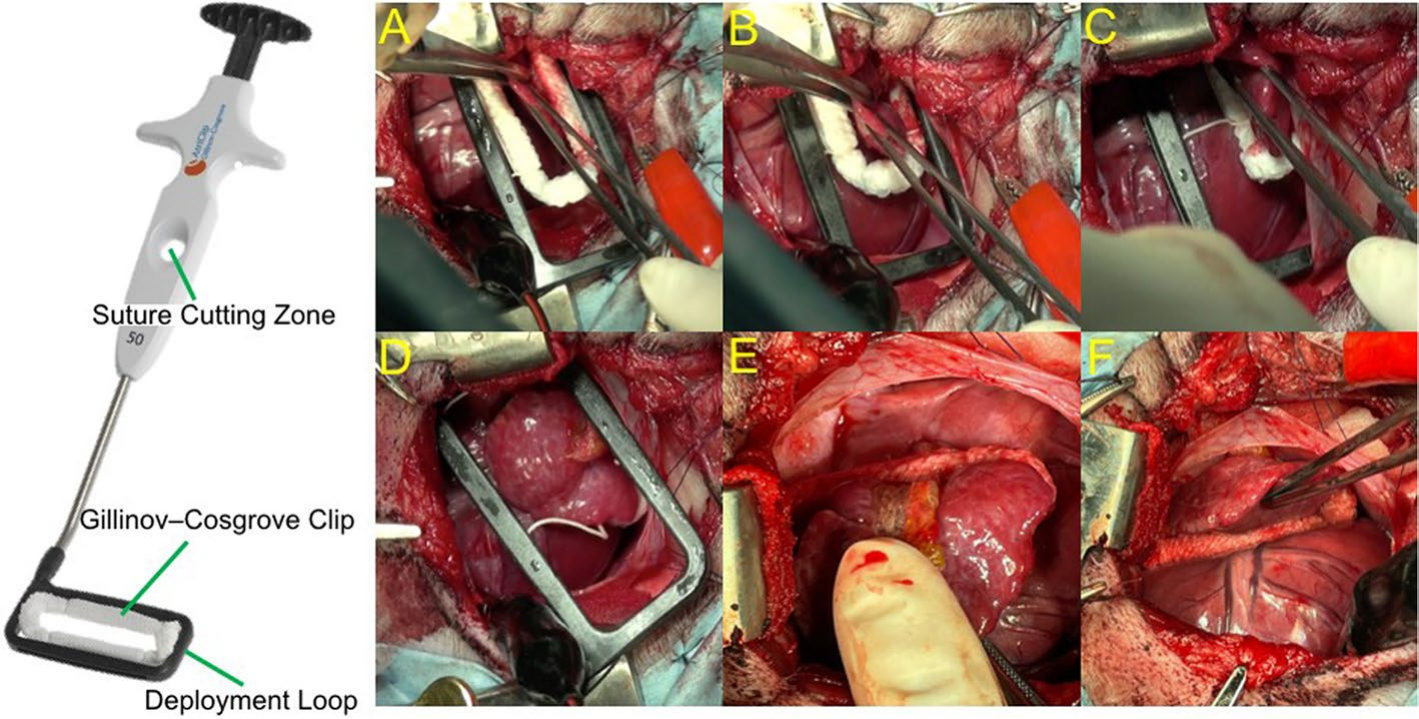
Atriclip for closure of the left atrial appendage. AtriClip consists of a Gillinov–Cosgrove Clip placed in a Deployment Loop. The Gillinov–Cosgrove Clip is made of two parallel titanium tubes with elastic nitinol springs covered by knit braided polyester. The nitinol springs apply a constant and durable force leading to closure of the left atrial appendage. Short-term stability is obtained by the force of the clip, and long-term durability is provided by tissue ingrowth. **a**, **b** Pull the left atrial appendage into the Gillinov–Cosgrove Clip. **c** Closure of the left atrial appendage. **d** Cutting off the string in Suture Cutting Zone. **e**, **f** After cutting off the thread, closing clip was detached from Deployment Loop

The patient was admitted to surgery 1 month after the initial presentation. Atropine sulfate (Mitsubishi Tanabe Pharma Corporation, Osaka, Japan, 0.05 mg/kg, subcutaneously), fentanyl (Fentanyl Citrate; Daiichi Sankyo Company, Limited, Tokyo, Japan, 5 μg/kg, IV), and midazolam hydrochloride (Dormicum; Astellas Pharma Inc., Tokyo, Japan, 0.2 mg/kg, SC) were administered as premedication. Anesthesia was induced with 1% propofol (Propofol Mylan; Mylan Seiyaku, Tokyo, Japan, 6 mg/kg bolus, IV) and maintained with 1–2 vol% of isoflurane (Isoflurane for Animal Use; Intervet, Osaka, Japan) and fentanyl (10 μg/kg/min) with 100% oxygen supplementation. The right femoral artery and vein were cannulated for invasive measurement of arterial and central venous blood pressures. After the patient was heparinized with 400 U/kg, the left carotid artery and jugular vein were cannulated for cardiopulmonary bypass. The mitral valve was approached through a fifth intercostal thoracotomy and left atrial incision. Cardiac arrest was achieved by aortic clamping followed by administration of cardioplegia (Miotecter; KYOWA CritiCare Co., Ltd., Tokyo, Japan) from the aortic root cannula. After cardiac arrest, the mitral valve was reconstructed by artificial chordae tendineae implantation and suture annuloplasty. Artificial chordae tendineae reconstruction and annuloplasty were performed with expanded polytetrafluoroethylene (ePTFE; GORE-TEX®, W. L. Gore & Associates, Inc., Newark, U.S.A.) suture as described in Fig. 2. After the closure of the left atrium, terminal warm coronary reperfusion and aortic declamping were performed. Sinus rhythm remained absent after declamping. Temporary pacing was attempted while atropine sulfate was administered. The patient did not have spontaneous electrical activity and was in cardiac arrest, and a decision was made for a permanent pacemaker programmed in VVI mode. Since the jugular vein was cannulated, the endocardial lead could not be placed. The pacemaker lead was placed on the left ventricular free wall and connected with the generator implanted subcutaneously in the abdomen behind the last rib on the left side. After implantation, the heartbeat depended on the pacemaker because the sinus node was not working. Left atrial appendage (LAA) closure with an AtriClip (AtriClip Left Atrial Appendage Exclusion System; AtriCure, Ohio, U.S.A.) was performed (Fig. 3). The patient was weaned from cardiopulmonary bypass after conducting modified ultrafiltration and later confirmed to be hemodynamically stable. A protamine infusion antagonized heparin, and the chest was closed in the usual manner.

**Fig. 4:**
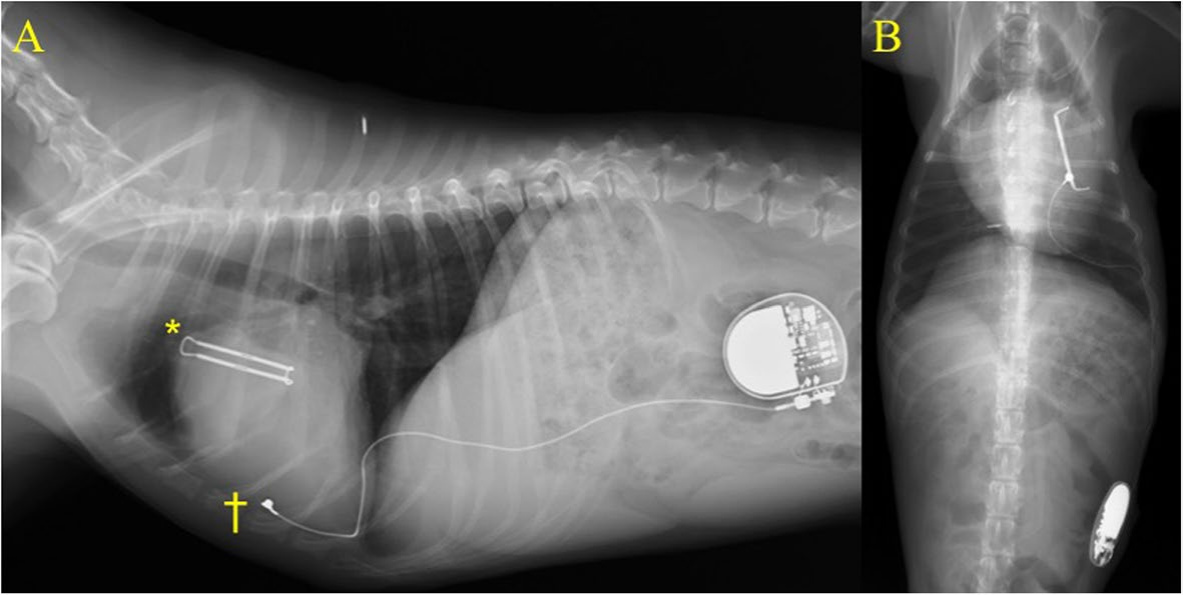
Thoracic radiography one month after surgery. Pacemaker lead and Atriclip were implanted in left ventricle and left atrial appendage. * shows Atriclip device. † shows pacemaker lead

Day 1 after surgery, the patient’s heart rate, and rhythm depended on the pacemaker (pacing rate: 80 bpm, programmed output: 3.5 V/0.4 ms, programmed sensitivity: 2.8 mV). Accelerated idioventricular rhythms with 140–170 bpm were frequently reported on Day 2. On Day 5, the heart rate depended mostly on the pacemaker. The patient was discharged on Day 11 with pimobendan 0.5 mg/kg, BID (Vetmedin; Boehringer Ingelheim, Ingelheim, Germany, 0.5 mg/kg, BID) and rivaroxaban 1.43 mg/kg, SID (Xarelto; Bayer Yakuhin, Ltd., Osaka, Japan). There was no clinical evidence of thrombosis during hospitalization. Echocardiographic parameters and thoracic radiography on discharge are presented in Table 1 and Fig. 4. After surgery, mitral regurgitation and the heart murmur mostly disappeared. E velocity and the left ventricular diameter decreased. At a follow-up visit on Day 30, the patient remained hemodynamically stable, and the owner reported no cardiac-related clinical signs. Echocardiography showed minimal mitral regurgitation with decreased E velocity and LVIDDN (Table 1, postoperative 1 month, and Fig. 1C, D). Electrocardiography showed an increased sinus rhythm; however, half of the heart rate depended on the pacemaker. All drugs were discontinued 3 months after surgery, and the patient continues in good health 10 months after surgery (Table 1, postoperative 10 months).

**Table 1:** Conventional echocardiographic parameters Preoperative and Postoperative-11 days, 1 Month, 2 Month, 3 Month in this case

Parameter	Pre OPE	Post OPE				
		11 days	1 month	2 month	3 month	10 month
BW, kg	7.55	7	7	7.5	7.7	7.7
HR	105	80	90	112	111	105
Echo variable						
LVIDd, mm	42.9	36.9	34.4	30.4	30.6	30.4
LVIDs, mm	25.3	25.5	25.9	20.9	22.7	20.9
LVIDDN	2.37	2.08	1.95	1.68	1.68	1.66
LA/Ao	2.84	1.83	1.82	1.68	1.62	1.68
FS, %	41	30.9	24.7	31.3	25.8	31.3
S′, cm/s	10.2	4.6	5.4	5.6	6.1	5.8
LVOT, cm/s	149	87	131	106	121	144
E velocity, cm/s	178	98	98	81	89	76
A velocity, cm/s	58	104	116	118	139	117
E/A	3.06	0.94	0.84	0.68	0.64	0.64

A velocity, peak velocity of late diastolic transmitral flow; BW, body weight; E/A, the ratio of peak velocity of early diastolic transmitral flow to peak velocity of late diastolic transmitral flow; E velocity, peak velocity of early diastolic transmitral flow, FS: fractional shortening; HR, heart rate; LA/Ao, the ratio of the left atrial dimension to the aortic annulus dimension; lat, mitral annulus at the left ventricular lateral wall; LVIDd, left ventricular internal dimension in diastole; LVIDDN, normalized left ventricular internal dimension in diastole; LVIDs, left ventricular internal dimension in systole; LVOT, left ventricular outflow tract; S′, systolic velocity of the LV wall (mean of free wall and septal wall)

## Discussion and conclusions

This paper reports an MVP procedure for a patient with MMVD and sinus dysfunction. A permanent pacemaker was implanted because of postoperative sinus dysfunction. Additionally, the LAA was closed with an AtriClip to avoid thrombus formation in the left atrium.

Patients in cardiac arrest during open-heart surgery occasionally require a pacemaker [11, 15]. Temporary pacemakers are used in emergency settings to treat arrhythmias after surgery [16]. Permanent pacemaker implantation is considered when mechanical pacing is required for more than a few days [11, 12]. This case report showed that dogs with impaired sinus node function might have difficulty restarting a beat and require mechanical pacing after mitral valve reconstruction surgery. Due to sinus arrest before surgery and the risk of the lead dislodging because of the patient’s movement, a permanent pacemaker was indicated. In this patient, the heart rate depended on the pacemaker for 5 days after surgery, indicating the importance of a pacemaker after surgery. The frequent presence of sinus rhythm documented during the follow-up visit indicates that postoperative mechanical pacing was critical for 2 to 4 weeks in this dog. For sick sinus syndrome (SSS) treatment, AAA mode is more physiological and induces better function. In this case, we should have chosen the pacemaker mode of AAA considering the pathophysiology of SSS; however, we performed pacemaker implantation using VVI pacing. VVI pacing was used during surgery to see if the atrial muscle responded to temporary pacing, but it did not; therefore, we chose VVI as a permanent pacemaker mode.

The incidences of postoperative complications such as thrombosis and arrhythmia are high and can be fatal [7, 8]. Decreased atrial myocardium mobility, often induced by severe arrhythmia such as atrial fibrillation, increases the incidence of intra-LAA thrombosis in human clinical settings [13]. Decreased LAA motility is thought to be the source of embolic strokes in up to 90% of cases, and occlusion of the LAA may be a useful method against coagulation [15, 16]. In this case, because of concomitant SSS, the atrium was hardly moving; thus, the risk of postoperative intra-atrial thrombosis was assumed high. Additionally, given the intrinsic procoagulant state after mitral valvuloplasty, LAA closure with an AtriClip was performed to reduce postoperative thrombosis. The advantages of LAA closure using an AtriClip are the simple and short-time technical process and the prevention of postoperative bleeding from the LAA. AtriClip is considered superior to suture or endocardial device closure. The application of an AtriClip also has some limitations. First, the device’s size; the AtriClip used in this case was 35 mm in clip length with a maximum device length of 50 mm, the smallest in the series. It is difficult to apply in small dogs that weigh less than 5 kg. Second, residual blood flow due to inadequate positioning is reported [17]. Good case selection and careful device placement can minimize these limitations.

In conclusion, in surgical cases of MMVD complicated with impaired sinus function, the chances are that spontaneous sinus rhythm will not return; thus, pacemaker implantation will be indicated. LAA closure may be considered against decreased atrial function after mitral valvuloplasty to prevent intra-atrial thrombus formation.

## Data Availability

The datasets used and/or analyzed during the current study are available from the corresponding author on reasonable request.

## Abbreviations

LVIDDN: Normalized left ventricular internal dimension in diastole
LAA: Left atrial appendage
MMVD: Myxomatous mitral valve disease
SSS: sick sinus syndrome
